# CD98 Positive Eosinophils Contribute to T Helper 1 Pattern Inflammation

**DOI:** 10.1371/journal.pone.0051830

**Published:** 2012-12-13

**Authors:** Fu-Min Xue, Huan-Ping Zhang, Hui-Jie Hao, Zhao-Yang Shi, Chuan Zhou, Baisui Feng, Ping-Chang Yang

**Affiliations:** 1 Department of Gastroenterology, The First Affiliated Hospital of Zhengzhou University, Zhengzhou, China; 2 Department of Pathology & Molecular Medicine, McMaster University, Hamilton, Ontario, Canada; Duke University Medical Center, United States of America

## Abstract

**Background and Aims:**

The pathogenesis of inflammatory bowel disease (IBD) has not been fully understood yet. Eosinophils (Eo) are one type of the major proinflammatory cells of the chronic inflammation in the intestine. CD98 is involved in the pathogenesis of a number of inflammations. This study aims to elucidate the role of CD98^+^ Eos in the initiation of intestinal inflammation.

**Methods:**

The colon biopsies were collected from 60 patients with IBD. The expression of CD98 in the biopsies was examined by immunohistochemistry. The serum levels of the flagellin (FGN) antibody and Eo-derived mediators in the culture supernatants were assessed by enzyme-linked immunosorbent assay. The role of FGN on Eo activation was examined in a cell culture model. The role of FGN in the induction of colitis was observed in a mouse model.

**Results:**

Compared to normal controls, the frequency of CD98^+^ Eos was markedly increased in the IBD colon mucosa. FGN were detected in the colon biopsies and in the sera of IBD patients. Exposure to FGN induced the expression of galectin 3 (the ligand of CD98) in dendritic cells. The exposure to galectin 3 activated the CD98^+^ Eos. After treatment with FGN intrarectally, mice with eosinophilia showed severe inflammation in the colon.

**Conclusions:**

The interaction of galectin 3 and CD98 can induce Eos to release chemical mediators that contributes to the initiation of the intestinal inflammation.

## Introduction

Inflammatory bowel disease (IBD) is a chronic inflammatory disorder of the intestine. The precise etiology of IBD is unclear. IBD includes two subtypes, ulcerative colitis (UC) and Crohn’s disease (CD). The inflammation in UC is limited to the mucosa of the colon, while CD may occur anywhere in the gastrointestinal tract that is characterized as transmural inflammation accompanying with or without granuloma in the intestinal tissue. The treatment of IBD is unsatisfactory currently [1].

Eosiniophils (Eos) contain a number of chemical mediators, such as Eo cationic protein (ECP), Eo peroxidase (EPO), Eo-derived neurotoxic protein (ENP) and major basic protein (MBP). Eos release chemical mediators upon activation [2]. The mediators are involved in the pathogenesis of allergic diseases such as asthma [3]. Besides, Eos are also involved in the pathogenesis of a number of other disorders, such as rheumatoid arthritis [4], infectious diseases [5] and idiopathic [6] inflammatory disorders. Eos normally distribute in the intestine with a plausible function to expel the parasitic infection [7]. The chemical mediators of Eos are involved in the intestinal inflammation. Yet, the mechanism by which Eos induce inflammation in the intestine remains to be further understood.

CD98 is a glycoprotein [8] that is encoded by two genes, the *SLC3A2* and *SLC7A5*. CD98 forms the large neutral amino acid transporter that is a heterodimeric membrane transport protein to transport branched-chain (valine, leucine, isoleucine) and aromatic (tryptophan, tyrosine) amino acids [9]. Intestinal epithelial cells express CD98 under the physiological conditions. Recent reports indicate that CD98 is involved in the intestinal inflammation, which can be upregulated by interferon (IFN)-γ in the intestine [8]. Others also indicate that CD98 is involved in the pathogenesis of colitis and cancer [9], [10]; the underlying mechanism is not fully elucidated yet.

Galectin-3 (Gal-3) is a member of the lectin family; it is encoded by a single gene, *LGALS3*, located on chromosome 14, locus q21–q22 [11]. Gal-3 is expressed by a number of cells. Published data indicate that Gal-3 is involved in the pathogeneses of cancer, inflammation, fibrosis, heart disease, and stroke. Gal-3 is the ligand of CD98 [12]. Dendritic cell (DC) express Gal-3 in response to appropriate stimulation [13]. Whether the interaction of DC-derived Gal-3 and CD98^+^ Eos plays any roles in the initiation of intestinal inflammation, such as IBD, is unclear. In the present study, we collected the colon biopsies from a group of IBD patients and found that Eos highly expressed CD98 in the IBD colon mucosa. DC-derived Gal-3 interacted with CD98 to activate Eos that contributed to the initiation of intestinal inflammation.

## Materials and Methods

### Reagents

Flagellin was purchased from Austral Biologicals (San Ramon, CA). Antibody of flagellin was purchased from Biodesign (Saco, Maine). Antibodies (S-17, C-terminus; N-20, N-terminus) and shRNA kits of CD98, TLR5 (B-24) and Gal-3 (H-5) were purchased from Santa Cruz Biotech (Santa Cruz, CA). Enzyme-linked immunosorbent assay (ELISA) kit for flagellin and rhGal-3 were purchased from R&D Systems (Shanghai, China). Anti-TLR5 antibody was purchased from Abcam (Cambridge, MA). Magnetic cell sorting reagents were purchased from Miltenyi Biotec (Auburn, CA). ELISA kits of EPO, EDN, MBP and ECP, neutralizing polyclonal antibodies of CD98 and Gal-3 were purchased from Uscn Life Science Inc. (Wuhan, China).

### Mice

BALB/c mice (6–8 weeks old) were purchased from the Beijing Experimental Animal Institute (Beijing, China). Mice were maintained in a pathogen-free environment. The experimental procedures were approved by the Animal Care Committee at Zhengzhou University.

### Human Subjects and Colon Biopsy

Sixty IBD patients (at remission stage) were recruited from the Department of Gastroenterology, Zhengzhou University and the China PLA General Hospital. The diagnosis of IBD was followed our routine procedures that were also published elsewhere [14], [15]. The colon biopsies were taken from the inflamed area of the intestine. The normal control tissue was obtained from the colon (non-polyp tissue) of 10 patients with colon polyposis. The demographic information of the IBD patients was listed in Table 1. The collection of colon biopsies was followed the established procedures in our departments. Informed, written consent was obtained from each patient. The study using human specimens in this study was approved by the Human Study Ethic Committees at Zhengzhou University and the China PLA General Hospital.

**Table 1:** Demographic and disease features of patients.

Group	Crohn’sdisease	Ulcerativecolitis	Colonpolyp
Sex	M: 15; F: 15	M: 15; F: 15	M: 5; F:5
Age	33.6 (28–66)	36.3 (23–74)	41.2 (18–61)
Weight (kg)	56.4 (51–75)	62 (54–77)	59 (55–71)
Duration of (months)	42 (26–65)	45 (34–86)	33 (12–45)
Prednisolone (mg/day)	15 (5–20)	15 (5–20)	0
Race	Asian	Asian	Asian
Outpatient	30	30	10
Previous hospitalizations	18	19	0
**Localization**			
Colon	30 (100%)	30 (100%)	10
Terminal ileum	6 (20%)		
Upper gastrointestinal	1 (3%)		
Left-sided Ulcerative colitis	0	4 (13.3%)	
Extensive Ulcerative colitis	0	3 (10.0%)	
**Behavior**			
Inflammatory	30 (100%)	30 (100%)	0
Stricturing	3 (10.0%)		
Penetrating	0		
Perianal disease	4 (13.3%),	4 (13.3%)	0
**Treatment**			
Steroids	30 (100%),	30 (100%)	0
Infliximab	5 (16.6%),	0	
Antibiotics	11 (36.7%),	14 (46.7%)	0
Mesalamine	3 (10.0%),	7 (23.3%)	0

Data are shown as medians (interquartile ranges).

### Serum Collection

Ten milliliter blood was collected from each IBD patient and 30 healthy subjects. The sera were isolated and stored at −80°C until use.

### Immunohistochemistry

The colon biopsies were frozen with liquid nitrogen. The cryosections were prepared and stained with antibodies of MBP (dilution: 1∶200) and CD98 (dilution: 1∶100) at 4°C overnight. The sections were then incubated with the horseradish peroxidase- or the alkaline phosphate-labeled secondary antibodies for 1 h at room temperature. The sections were finally stained with haematoxylin and observed with a light microscope. All the sections were coded; the observers were not aware of the code to avoid bias. The positively stained cells were counted in twenty fields (×400) per sample.

### ELISA

The serum levels of flagellin antibody, EPO, EDN, MBP and ECP were determined by ELISA with commercial reagent kits following the manufacturer’s instructions.

### Generation of Human DCs

Twenty healthy volunteers were selected as blood donors. Peripheral blood mononuclear cells (PBMCs) were separated using the Histopaque (SG-1.077; Sigma) density gradients. The CD11c^+^ DCs were separated from the PBMCs by magnetic cell sorting (MACS). As checked by flow cytometry (FACS), the purity of the separated DCs was over 90%. The cells were cultured in RPMI 1640 medium containing 10% fetal calf serum, 2 mM l-glutamine, 100 U/ml penicillin, 100 µg/ml streptomycin, 25 mM HEPES (4-(2-hydroxyethyl)-1-piperazineethanesulfonic acid ) and Granulocyte-macrophage colony-stimulating factor (20 ng/ml) under 37°C in 5% CO_2_ environment. Three days later, the cells were collected and used for further experiments.

### Quantitative Real-time RT-PCR (qRT-PCR)

The expression of Gal-3 in DCs was determined by qRT-PCR. Twenty-five ng cDNA were amplified in 50 µl 1 × SYBR-Green I PCR master mix, containing 200 nM primers for Gal-3. The experiments were performed in triplicate using a qRT-PCR thermal cycler (Bio-Rad). The negative controls (master mix containing untranscribed total RNA or without any cDNA or RNA) were added to each experiment. The quantification was obtained using the relative standard curve method. The standard curve was created by amplification of 5 µl control total RNA extracted from the cells at different dilutions (1.0–0.025 ng/µl by twofold dilution). The relative amount of each unknown sample was calculated using the linear regression from the standard curve. The relative Gal-3 gene expression value was normalized to percentage of the value of the housekeeping gene, β-actin. The primers of Gal-3 using in this study were: Forward: ggccactgattgtgccttat; reverse: gaagcgtgggttaaagtgga (NCBI: NM_001177388.1).

### Western Blotting

A piece of the intestinal mucosa was processed for protein extraction. Protein extracts were subjected to electrophoresis on a 10% SDS-polyacrylamide electrophoresis gel. The proteins were transferred onto nitrocellulose membrane, which was then blocked with 5% bovine serum albumin (BSA) in Tris-buffered saline containing 0.05% Tween 20 (TBST) for 1 h. The blot was then incubated with the primary antibody (1∶500) overnight at 4°C in 5% BSA/TBST. The blot was washed with TBST and further incubated with the horseradish peroxidase-conjugated secondary antibody (1∶1000) for 1 h at room temperature. Subsequently, the blot was washed 3 times with TBST for 1 h, and developed with SuperSignal chemiluminescence reagent, and exposed to X-ray film to visualize the proteins.

### Culture of Eo Cell Lines

The Eo cell line, EoL-1 cells, generated from a patient with acute myeloid leukemia following hypereosinophilic syndrome, was purchased from the RIKEN Cell Bank (Tsukuba, Japan). EoL-1 cells were maintained in RPMI 1640 medium supplemented with 10% fetal calf serum and antibiotics at 37°C and 5% CO_2_.

### RNA Interference

The gene expression of TLR5 in DCs and CD98 in EoL cells was knocked down by the transduction with shRNA reagent kits for TLR5 and CD98 respectively. The experiments were performed following the manufacturer’s instruction. The efficiency of gene knockdown was over 90% as assessed by Western blotting (data not shown).

### Induction of Intestinal Eosinophilia

A parasitic infection model was adopted to induce eosinophilia in the intestine. The maintenance of the *T. muris* parasite and the methods using for infection and large intestinal worm burden assessment were performed as previously described [16]. BALB/c mice were infected by oral gavage with 120–150 infective eggs/mouse and sacrificed or used for further experiments 45 days later. The cecum was removed and opened to count the number of parasites. The results showed the parasites were found in the cecum of all infected mice (5–11 parasites were found in each cecum. The colon was excised and processed for paraffin sections and stained with the H&E method. The number of Eos was counted under a light microscope. The results showed that the number of Eos was 38.5±5.8/mm^2^ in infected mice in contrast to 9.6±2.5/mm^2^ in saline control mice (p<0.01).

### Induction of Colitis in Mice with Eosinophilia

BALB/c mice with intestinal eosinophilia (the eosinophilic status in the intestine was verified by examining sample mice) were intrarectally introduced with 0.1 ml 50% ethanol containing FGN 50 µg/mouse (control mice were introduced with ethanol alone) under light general anesthesia, twice a week for 3 weeks. The body weight of each mouse was recorded before the treatment and before the sacrifice. The mice were sacrificed by cervical dislocation. A segment of the colon was excised and processed for paraffin embedding and H&E staining. A piece of the colon was used to extract protein to measure the levels of myeloperoxidase (MPO).

### DC Depletion

A group of mice was depleted DCs by ip injection with dichloromethylenediphosphonic acid-loaded or PBS-loaded liposomes (Encapsula NanoSciences) or intravenously into mice (200–300 µl per mouse) as previously described [17]. As checked by immunohistochemistry, CD11c^+^ DCs were not observed in the intestine of the mice (data not shown).

### Depletion of CD98 and Gal-3 in Mice

BALB/c mice were ip injected with neutralizing anti-CD98 antibody (0.2 mg/mouse) or neutralizing anti-Gal-3 (0.2 mg/mouse) antibody daily for 5 consecutive days. As shown by the results of Western blotting, the expression of CD98 or Gal-3 was below the detectable levels in the intestine of the mice (data not shown).

### Measurement of MPO Levels

A piece of the colon tissue was excised and homogenized in lysis buffer (200 mM NaCl, 5 mM EDTA, 10 mM Tris, 10% glycerin, 1 mM PMSF, 1 µg/mL leupeptin, and 28 µg/mL aprotinin (pH 7.4); 200 µL). Samples were centrifuged at 15000 ×g at 4°C for 15 minutes. The supernatants were collected; the MPO activity was assayed by mixing the supernatants with citric phosphate buffer (pH 5.0) containing 0.4 mg/ml O-phenylene diamine and 0.015% hydrogen peroxide. The optical density value of the samples were measured with a spectrophotometer at 450 nm.

### Statistics

The values were expressed as the means with the SEM of at least three independent experiments. The data were analyzed using the two-tailed unpaired Student t test when the data were consisted of two groups or by ANOVA when three or more groups were compared. A value of p<0.05 was accepted as statistically significant.

## Results

### Frequency of CD98+ Eos was Increased in IBD Intestinal Mucosa

**Figure 1:**
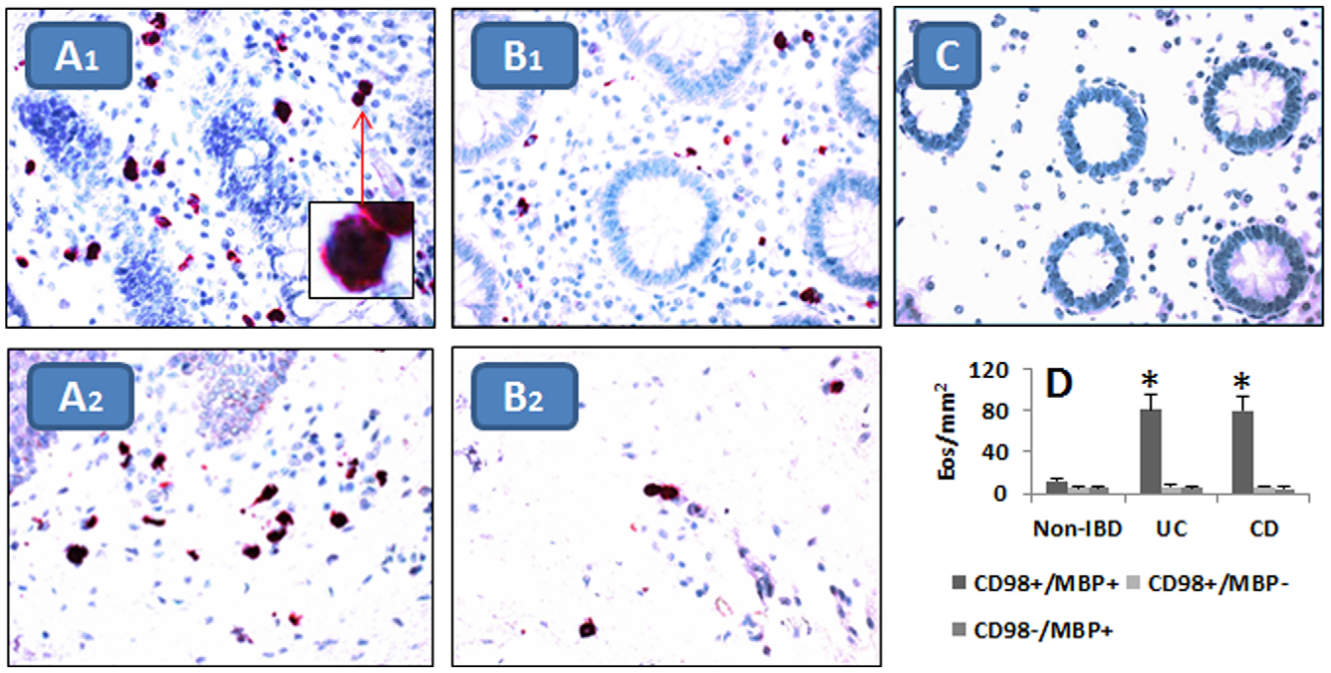
Intestinal Eos express CD98. Colon biopsies were obtained from 60 IBD patients (30 UC; 30 CD). The bioptic specimens were processed for cryosections and stained with antibodies of CD98 and MBP by a combination of peroxidase-nickel and alkaline phosphate methods. A-B, representative images show CD98^+^ (in red) and MBP^+^ (in black) Eos in the colon mucosa of IBD (A) and non-IBD (B; from 6 patients with colon polyps). Original magnification: ×400. Panel C is an isotype control. D, the bars indicate the frequency of CD98^+^ or/and MBP^+^ Eos (mean ± SEM). *, p<0.01, compared with non-IBD samples. The sections were contrast-stained with hematoxylin (no eosin staining). A1 and B1 were stained with anti-CD98 C-terminus antibody; A2 and B2 were stained with anti-N-terminus antibody.

Previous studies suggested that the CD98 might play a role in the pathogenesis of IBD [9], [10]. In the present study, we observed that the expression of CD98 was not only expressed on the epithelial cells, but also expressed on some cells in the lamina propria; most of the CD98^+^ cells are polynuclear cells. The staining with eosin revealed that the cells were Eos. Immune staining revealed that more than 80% CD98^+^ cells in the lamina propria were Eos (MBP^+^); the frequency of CD98^+^ Eos was not significantly different between UC and CD (Fig. 1). Eos were also detected in the non-IBD intestinal mucosa; but the frequency (11.6% ±2.2%;) of CD98^+^ Eos was significantly lower in the non-IBD specimens than that in the IBD intestinal mucosa.

### Flagellin Induces Expression of Gal-3 in DCs

**Figure 2:**
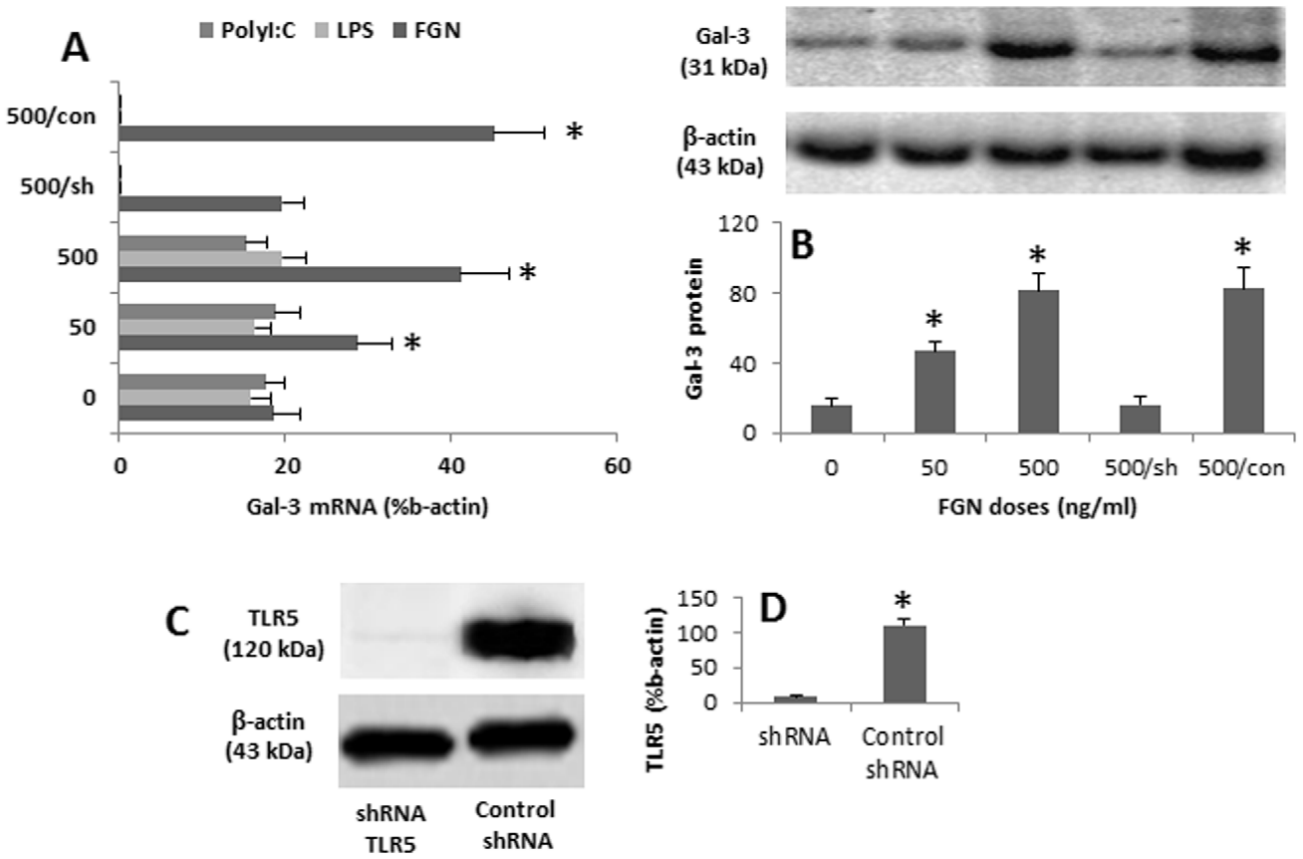
FGN induces the expression of gal-3 in DCs. DCs were generated from human PBMC and cultured in the presence of FGN, or LPS, or PolyI:C, at the indicated doses for 48 h. The expression of gal-3 in DCs was determined by qRT-PCR (A) and Western blotting (B). A, the bars indicate the mRNA levels of Gal-3 that were normalized to the percentage of β-actin. B, the immune blots shows the levels of Gal-3. The bars below the immune blots indicate the summarized integrated density of the immune blots. The data in bar graphs were expressed as mean ± SEM. *, p<0.05, compared with dose “0″ group. sh: DCs were pre-treated with shRNA of TLR5 (for FGN group) for 48 h before using in the experiments. Con: DCs were pre-treated with control shRNA. C–D, the immune blots show the levels of TLR5 in DCs treated with shRNA of TLR5 or control shRNA (C); the bars indicate the summarized integrated density of the immune blots (D). The data represent 6 experiments.

We next investigated the ligands of CD98. Gal-3 is the ligand of CD98 [12]. DCs express Gal-3 in response to appropriate stimulation [13]. DCs may be activated by the interaction of microbial products and Toll-like receptors (TLR). We then prepared DCs and exposed the DCs to microbial products, FGN, PolyI:C and LPS in the culture. The expression of Gal-3 in DCs was examined. The results showed that the exposure to FGN in the culture increased the expression of Gal-3 on DCs in a FGN dose-dependent manner while the exposures to LPS or PolyI:C did not. To confirm the results, we knocked down the TLR5 in DCs by the gene silence; the TLR5-deficient DCs were then exposed to FGN in the culture. As expected, the increase in the expression of Gal-3 was abolished (Fig. 2). The results indicate that exposure to FGN can upregulate the expression of Gal-3 on DCs.

### DC-derived Gal-3 Activates CD98^+^ Eos

**Figure 3:**
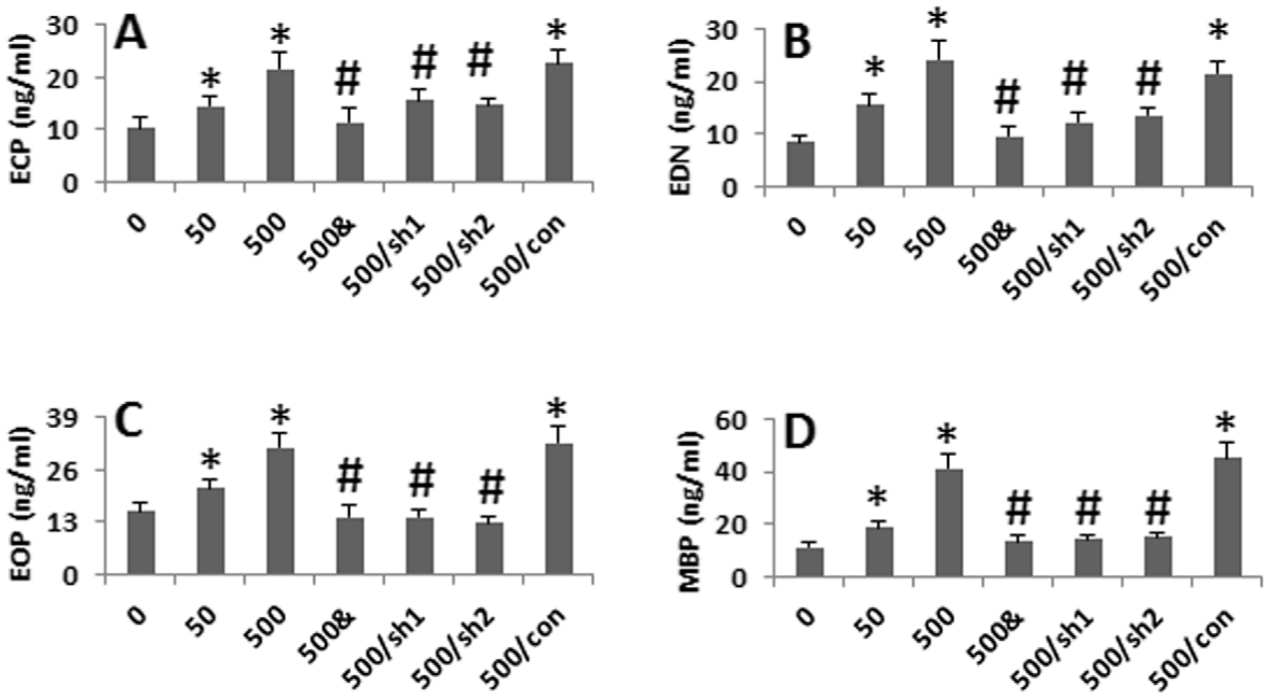
Levels of the Eo-derived chemical mediators in culture supernatant. DCs and EoL cells were cultured in the presence of FGN at graded doses for 24 h. The bars indicate the levels of the Eo-derived mediators that were determined by ELISA. The data were presented as mean ± SEM. *, p<0.05, compared with group “0″. #, p<0.01, compared with group “500”. &: No DCs were added to the culture. sh1: EoL cells were pretreated with the shRNA of CD98. sh2: DCs were treated with shRNA of Gal-3. Con: EoL cells were pretreated with the control shRNA. Super: EoL cells were exposed to the supernatants of DC treated with FGN (500 ng/ml; 12 h). rhGal3: EoL cells were stimulated with rhGal-3. The data represent 6 experiments.

As shown in Fig. 1, high frequency of CD98^+^ Eos was observed in the IBD intestinal mucosa; Gal-3 is the ligand of CD98 [12], we inferred that the interaction of Gal-3 and CD98 might activate Eos. To test the hypothesis, we cultured DCs and an Eo cell line, the EoL-1 cells, in the presence of FGN for 24 h. Then, the levels of the Eo-derived chemical mediators in the culture supernatant were assessed by ELISA. The results showed that the exposure to FGN markedly increased the levels of the chemical mediators, including ECP, EPO, EDN and MBP, in the supernatant, that was abolished by knocking down the gene of CD98 in EoL-1 cells or in the absence of DCs. Cultured with the supernatant of FGN-treated DCs or addition of recombinant Gal-3 to the culture also increased the Eo-derived mediators in the culture supernatant (Fig. 3). The results indicate that the interaction of Gal-3 and CD98 can activate Eos to release proinflammatory mediators.

### Serum Levels of FGN Ab in IBD Patients are Correlated with the Frequency of CD98^+^ Eos

**Figure 4:**
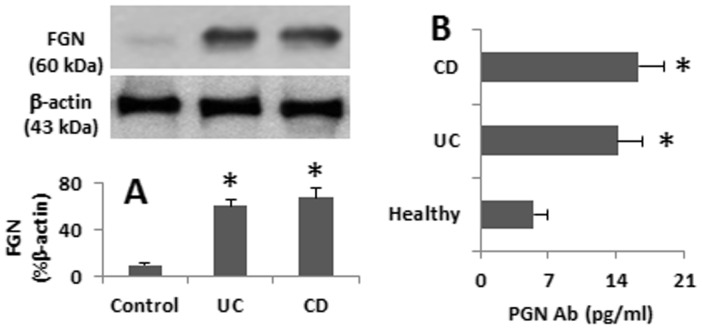
The levels of FGN antibody (Ab). A, one piece of the biopsies from each IBD patient was processed for Western blotting. The representative immune blots show FGN in the colon tissue. The bars show the summarized integrated density of the immune blots [mean ± SEM from 60 IBD specimens and 10 controls (the colon biopsies were obtained from patients with colon polyps)]. B, the sera were collected from 30 UC patients, 30 CD patients and 30 healthy subjects. The levels of FGN Ab were determined by ELISA. The bars indicate the levels of serum FGN Ab. The data were presented as mean ± SEM. *, p<0.01, compared with healthy controls.

Prompted by the results of Fig. 2 and Fig. 3, we measured the serum levels of FGN antibody in 60 IBD patients and 30 healthy subjects. As shown by ELISA, the serum levels of FGN Ab were significantly higher in IBD patients than that in healthy controls (Fig. 4; p<0.01). Furthermore, a positive correlation was identified between the frequency of CD98^+^ Eos in the intestinal biopsies and the serum levels of FGN Ab (r = 0.548, p<0.05). In addition, FGN was detected in the colon biopsy specimens by Western blotting that was markedly higher in IBD samples than that in controls (10 patients with colon polyps).

### Exposure to FGN Induces Colitis in Mice with Intestinal Eosinophilia

**Figure 5:**
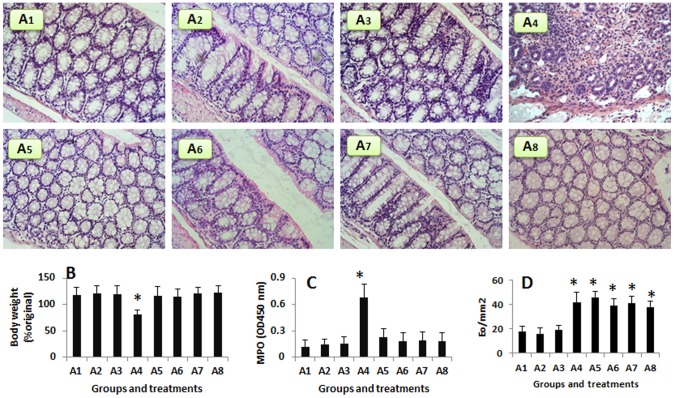
Exposure to FGN induces inflammation in the colon of mice with eosinophilia. Mice were induced the eosinophilia in the intestine by parasitic infections. Naïve mice (A1) did not have any specific treatment. A2–A3, naïve mice were treated with saline (A2) or FGN (A3) via the intrarectal route. A4–A7, mice with eosinophilia were exposed to FGN via intrarectal route and depletion of DCs (A5), or anti-CD98 antibody (A6), or anti-Gal-3 antibody (A7). A8, mice with intestinal eosinophilia were treated with 50% ethanol via the intrarectal route. Image magnification: ×200. B, the bars indicate the body weight records; the data were presented as a percentage of the original body weight. C, the bars indicate the MPO level in colon tissue extracts. D, the bars indicate the frequency of Eo in the colon mucosa (the counts were averaged from 20 fields per mouse). The data in B-D were expressed as mean ± SEM. *, p<0.05, compared with group A1. Each group had 6 mice.

**Figure 6:**
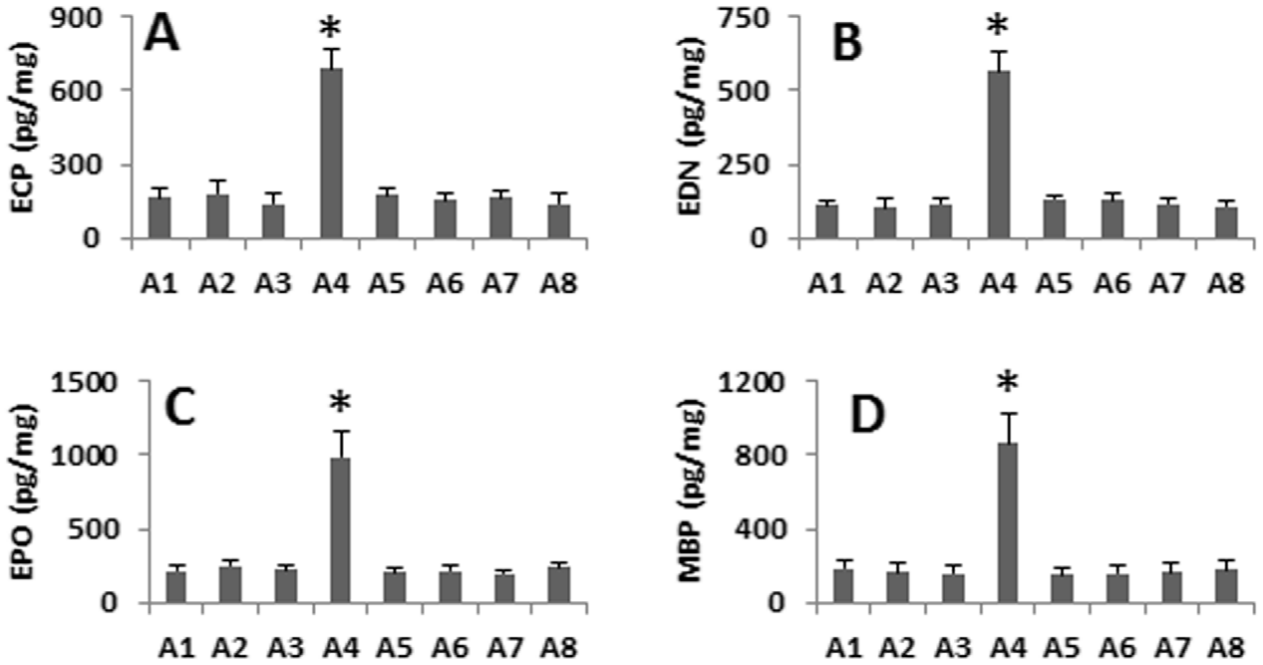
Levels of Eo-derived mediators in the intestine. The sera were collected from the mice in Fig. 5 and were analyzed for the levels of Eo-derived mediators by ELISA. The bars indicate the levels of ECP (A), END (B), EPO (C) and MBP (D). The data were presented as Eo mediators/mg serum protein (mean ± SEM from 6 mice per group). The X axes list the groups that are the same as those in Fig. 5. *, p<0.01, compared to the A1 group. Each group consisted of 6 mice.

Finally we developed the eosinophilia in the mouse intestine by the parasitic infection. The mice were intrarectally introduced with FGN for 5 days. The histology of the colon showed that colitis was induced manifesting abundant inflammatory cell infiltration, tissue edema, intra-tissue bleeding and deformation of crypts (Fig. 5A). High frequency of Eos was also observed in the intestine (Fig. 5A). In addition, the body weight loss (Fig. 5B), high levels of MPO of the colon extracts (Fig. 5C) and bleeding stool were recorded. These pathological changes were abolished in mice pretreated with either anti-CD98 antibody, or anti-Gal-3 antibody, or depletion of DC (Fig. 5D). The serum levels of Eo-derived mediators were significantly higher only in mice with eosinophilia and exposure to FGN (Fig. 6).

## Discussion

The present paper reports that the intestinal Eos express CD98, which is significantly higher in the intestinal mucosa of patients with IBD that non-IBD intestinal mucosa. The frequency of CD98^+^ Eos is positively correlated with the serum level of FGN antibody in IBD patients. The data also indicate that FGN can induce DCs to release Gal-3; the latter ligates CD98 on Eos to induce Eos to release the chemical mediators, the latter contribute to the initiation of inflammation in the intestine. Thus, in addition to the well characterized IL-5 on activation of Eos [18], the present data provide a novel pathway to activate Eos in the intestine.

Eos are the residential cells in the intestine. The cells contribute to the innate immunity by inhibiting parasitic infection in the intestine [2]. Because of containing a number of chemical mediators, it is suggested that the activation of Eos is involved in the initiation of inflammation in the intestine. High levels of Eo-derived mediators can be detected in feces [19] or colon perfusion fluids [20] of patients with chronic colitis that are positively correlated with the infiltration of Eos in the colon. The present data are in line with these reports by showing a significant increase in the frequency of Eos in IBD intestinal biopsies.

A novel finding of the present study is that Eos express CD98. The data show that the CD98^+^ Eos were detected in both IBD colon biopsies and non-IBD colon tissue; the frequency of CD98^+^ Eos was significantly higher in the IBD specimens than that in the non-IBD samples. The underlying mechanism might be that there is a high level of IFN-γ in IBD intestinal tissue; IFN-γ can upregulate the expression of CD98 [9], which may induce Eos to express high levels of CD98. A recent report [10] indicates that the over-expressing CD98 results in inflammation in the intestine or promotes the carcinogenesis in the intestine. To date, previous reports only mentioned that intestinal epithelial cells express CD98. In line with the report [10], our results not only show that the expression of CD98 in the intestinal epithelial layer (data not shown), but also reveal a high frequency of CD98^+^ Eos in the intestine of patients with IBD. The present data show that the activation of CD98^+^ Eos can compromise the epithelial barrier function, which results in more macromolecular antigens to be absorbed into the deep tissue of the intestine. The data further demonstrate that the activation of CD98 causes Eos to release the chemical mediators; the mediators may attribute to the inflammation in IBD. The inference is supported by the subsequent data from the animal model study. The exposure to FGN induced severe colitis in mice with eosinophilia. Previous study also demonstrated that FGN was associated with intestinal inflammation [21]. Although several control groups, including those treated with anti-CD98, anti-Gal-3 and ethanol, also had intestinal eosinophilia, those mice did not have apparent signs of colitis. The fact indicates that the increase in the Eo number alone is insufficient to cause inflammation in the intestine until the Eos are activated somehow. Indeed, the serum levels of Eo-derived chemical mediators, one of the signs of Eo activation, were markedly increased in mice with eosinophilia and exposure to FGN but not in those control mice.

Gal-3 is the ligand of CD98 [12]. The expression of Gal-3 in macrophages has been well-documented [13]. Following published procedures [13], we also generated Gal-3 expressing DCs. The generated DCs express TLR5; the fact indicates that these DCs can recognize the FGN’s stimulation. Our further data proved the inference by showing that exposure to FGN, abundant Gal-3 were detected in the DCs as well as in the culture supernatants. Gal-3 is also an adhering molecule that facilitates the Eo rolling and plays a role in allergic inflammation, cancer, inflammation and fibrosis, heart disease, and stroke [22]–[24]. Our data also demonstrate that Gal-3 is involved in facilitating the development of inflammation by showing an indirect effect that the interaction of Gal-3/CD98 induces Eos to release chemical mediators; the latter induces inflammation in the intestine.

In the present study, the eosinophilia and the presence of FGN are important factors in the induction of colitis. However, eosinophilia is common in the parasite infection; FGN is also a regular microbial product in the intestine, not all patients with parasite infections suffer from IBD. In the present study, that mice with eosinophilia were prone to colitis is based on a premise of breaking the epithelial barrier function after treating with ethanol. The fact emphasizes that the integrity of intestinal barrier function is important to maintain the homeostasis in the intestine. Our results are in line with a recent report that intestinal epithelial cells express CD98 is involved in the induction of colitis in mice [10]. A difference between our data and Nguyen’s study [10] is that we observed that intestinal Eos expressed CD98 while they revealed that intestinal epithelial cells expressed CD98. The fact indicates that more than one cell types express CD98 in the intestine, which may be associated with the pathogenesis of intestinal inflammation.

To further confirm the role of CD98 in the induction of the intestinal inflammation in the present experimental system, we pretreated a group of mice with anti-CD98 antibody, which blocked the inflammation. However, as shown by histology, the number of Eos was not decreased in this group of mice. The fact indicates that administration with anti-CD98 did not cause the death of Eos in the intestine; only neutralized the CD98 on Eos. The underlying mechanism is to be further investigated. In fact, Eos are not the only cell type expressing CD98, many other cell types [25] also express CD98. Whether the anti-CD98 antibody interfered with other cells’ function was not systematically observed, which needs to be further investigated.

In summary, our data indicate that Eos express CD98 in IBD intestinal tissue. Microbial product, FGN, can activate DCs to express Gal-3. The Gal-3 binds CD98 to activate Eos to release chemical mediators and induces inflammation in the intestine.
